# ﻿*Mesochra vietnamica* sp. nov. (Copepoda, Harpacticoida, Canthocamptidae), the first record of the genus from Vietnam, with a key to Asian species

**DOI:** 10.3897/zookeys.1265.174622

**Published:** 2025-12-29

**Authors:** Chaichat Boonyanusith, Thi-Phuong Pham, Minh Anh Dam, Ngoc-Son Tran, Samuel Gómez

**Affiliations:** 1 School of Biology, Faculty of Science and Technology, Nakhon Ratchasima Rajabhat University, Nakhon Ratchasima, 30000, Thailand Nakhon Ratchasima Rajabhat University Nakhon Ratchasima Thailand; 2 The University of Danang - University of Science and Education, 459 Ton Duc Thang St., Danang City 550000, Vietnam The University of Danang - University of Science and Education Danang Vietnam; 3 Universidad Nacional Autónoma de México, Instituto de Ciencias del Mar y Limnología, Unidad Académica Mazatlán, Joel Montes Camarena s/n, Mazatlán, 82040, Sinaloa, Mexico Universidad Nacional Autónoma de México Mazatlán Mexico

**Keywords:** Mangrove, new species, Southeast Asia, taxonomy

## Abstract

A new species of the genus *Mesochra* Boeck, 1865 was collected from mangrove habitats at the estuary of the Vu Gia–Thu Bon river system, central Vietnam. *Mesochra vietnamica***sp. nov.** is morphologically close to *M. oligochaeta* Kornev & Chertoprud, 2008, but differs in i) the position of the inner seta on P1 Enp-1, ii) the length/width ratio of P1 Enp-1, iii) the number of elements on the antennary exopod, and iv) the number of setae on P2 Enp-2. The new species belongs to the group of congeners with P1 Enp-1 longer than the entire exopod. A key to the Asian species of *Mesochra* is provided.

## ﻿Introduction

The genus *Mesochra* Boeck, 1865 was established by Boeck (1865) for *M. lilljeborgii* Boeck, 1865, *M. kroyeri* Boeck, 1865, and *M. pygmaea* (Claus, 1863). Lang (1948) recognized 16 species of *Mesochra*. Hamond (1971) validated 31 species and designated *M. lilljeborgii* as the type species. Fiers and Rutledge (1990) recognized 34 species, and the number of accepted species increased to 36 with the addition of *M. pacifica* Gómez-Noguera & Fiers, 1997 and *M. pseudoparva* Gómez-Noguera & Fiers, 1997. Gómez and Steinarsdóttir (2007) presented the description of three species from Iceland and gave an updated historical account of the genus. Wells (2007) considered *M. reducta* Klie, 1950 as a *species inquirenda*. Three more species were subsequently added from Russia (Kornev and Chertoprud 2008), South Korea (Lee and Chang 2008) and Colombia (Suárez-Morales and Fuentes-Reinés 2015). In addition, Alper et al. (2023) transferred *M. armoricana* to *Nannomesochra* Gurney, 1932. According to Walter and Boxshall (2025), the genus is currently composed of 44 accepted species and five subspecies.

The genus is one of the largest genera of the family Canthocamptidae and comprises species that primarily inhabit brackish environments. Most other members of the family, however, are predominantly found in freshwater habitats (Boonyanusith et al. 2024; Tran et al. 2025). Fifteen species have been recorded from Asia (Table 1). Among them, two species are known from Southeast Asia: *M. prowazeki* van Douwe, 1907 from Sumatra (Indonesia) (van Douwe 1907), and Mesochra cf. pygmaea from Nha Trang Bay (Vietnam) (Chertoprud et al. 2009).

**Table 1.** Species of *Mesochra* recorded in Asia.

Taxa	Localities	References
*Mesochra aestuarii* Gurney, 1921	Aral Sea and Mouth of Amu Darya River (Turkestan^?^)	Borutzky (1927)
*Mesochra aralensis* Borutzky, 1927	Aral Sea (Turkestan^?^)	Borutzky (1927)
*Mesochra alaskana* Wilson, 1958	Hokkaido (Japan), South Korea	Ishida (1987); Ishida and Kikuchi (2000); Lee and Chang (2003)
*Mesochra bisetosa* Lee & Chang, 2008	Imjado Island (South Korea)	Lee and Chang (2008)
*Mesochra hinumaensis* Kikuchi, 1972	Lake Hinuma (Japan), South Korea	Kikuchi (1972); Chang (2010)
*Mesochra meridionalis* Sars, 1905	Chingrighata (India)	Sars (1905); Sewell (1934)
*Mesochra nana* Brady, 1910	Chilka Lake (India)	Sewell (1924)
*Mesochra prowazeki* Douwe, 1907	Tianjin (China), Sumatra (Indonesia)	van Douwe (1907); Shen et al. (1979)
Mesochra cf. pygmaea (Claus, 1863)	Nha Trang Bay (Vietnam)	Chertoprud et al. (2009)
*Mesochra quadrispinosa* Shen & Tai, 1965	Guangdong and Fujian (China)	Shen and Tai (1965)
*Mesochra rapiens* (Schmeil, 1894)	Shimushu Island and Hokkaido (Japan)	Chappuis (1932); Ishida (1987)
*Mesochra sewelli* Lang, 1948	Chilka Lake (India)	Lang (1948)
*Mesochra suifunensis* Borutzky, 1952	Wuli Lake (China), South Korea	Shen et al. (1979); Lee and Chang (2003)
*Mesochra wolskii* Jakubisiak, 1933	Vijayawada city and Tamil Nadu (India)	Ranga Reddy (2014); Wells (1971)

^?^ former name of the area in which the locality is located.

A new species of the genus was discovered during our continuing investigations of brackish copepods in mangrove forests from central Vietnam. Herein we present the full description of the new species and a key to the species known from Asia.

## ﻿Materials and methods

Plankton samples were taken from the mangrove zone at the mouth of Vu Gia–Thu Bon River (Da Nang City, central Vietnam) by horizontal trawls at the depth of 1 meter below the water surface with a 50 μm plankton net. The collected specimens were immediately preserved in 70% ethanol. Individuals were sorted at the laboratory using a stereomicroscope at 40× magnification. Dissected appendages were mounted in a drop of pure glycerol on glass slides and then sealed with transparent nail varnish. Undissected specimens were stored in 70% ethanol for future taxonomic reference (Dussart and Defaye 2001).

Observations of body structures and ornamentation were carried out using a Carl Zeiss Axio Lab A1(Germany) compound microscope at 400–1000× magnification. Digital images were acquired as stack microphotography and combined with GIMP v. 3.1.4 to produce single photo from different depths of focus. Line drawings of the body and the appendages were made digitally using CorelDRAW v. 19.0.

The descriptive terminology follows Huys and Boxshall (1991). The abbreviations in the text and tables are as follows: ae = aesthetasc; Enp = endopod; Exp = exopod; Exp/Enp-1(2, 3) = proximal (middle, distal) segment of the exopod or endopod; and P1–P6 = the first to sixth swimming legs.

The type material has been deposited at the Zoological Collection of Duy Tan University (**ZC-DTU**>) in Da Nang City, Vietnam.

## ﻿Taxonomy

**Order Harpacticoida Sars, 1903**

**Family Canthocamptidae Brady, 1880**

**Genus *Mesochra* Boeck, 1865**

### ﻿ Mesochra vietnamica sp. nov.

Taxon classificationAnimaliaHarpacticoidaCanthocamptidae

419AFFD8-0FF6-5E5E-830A-4C4A1982D1D7

https://zoobank.org/2313CFB5-8720-4A51-B20C-6B8F414BDA8F

**Figure 1.**
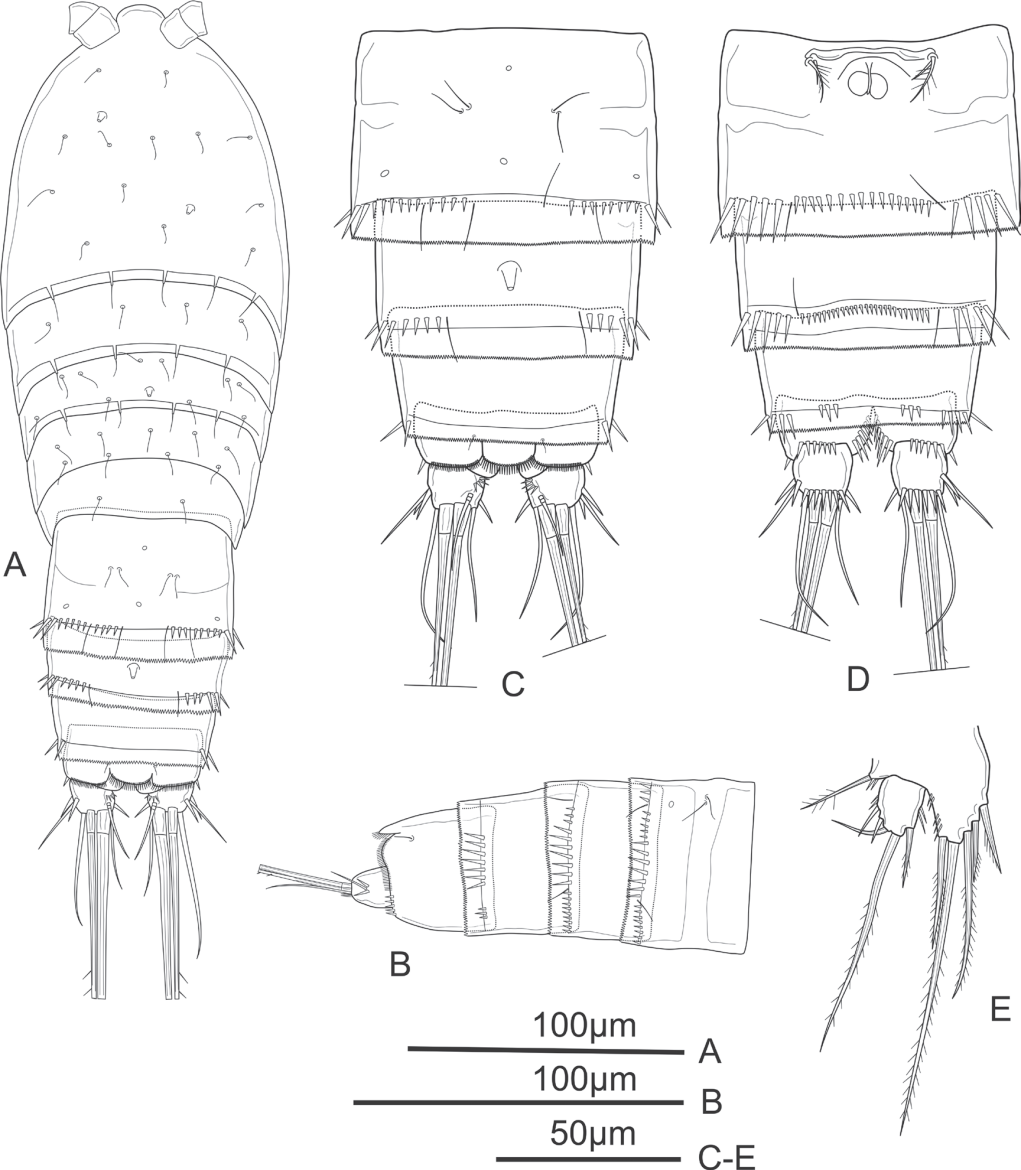
*Mesochra vietnamica* sp. nov. Female, holotype. A. Habitus, dorsal view; B. Urosome (P5-bearing somite omitted), lateral view; C. Urosome (P5-bearing somite omitted), dorsal view; D. Urosome (P5-bearing somite omitted), ventral view; E. P5.

**Figure 2.**
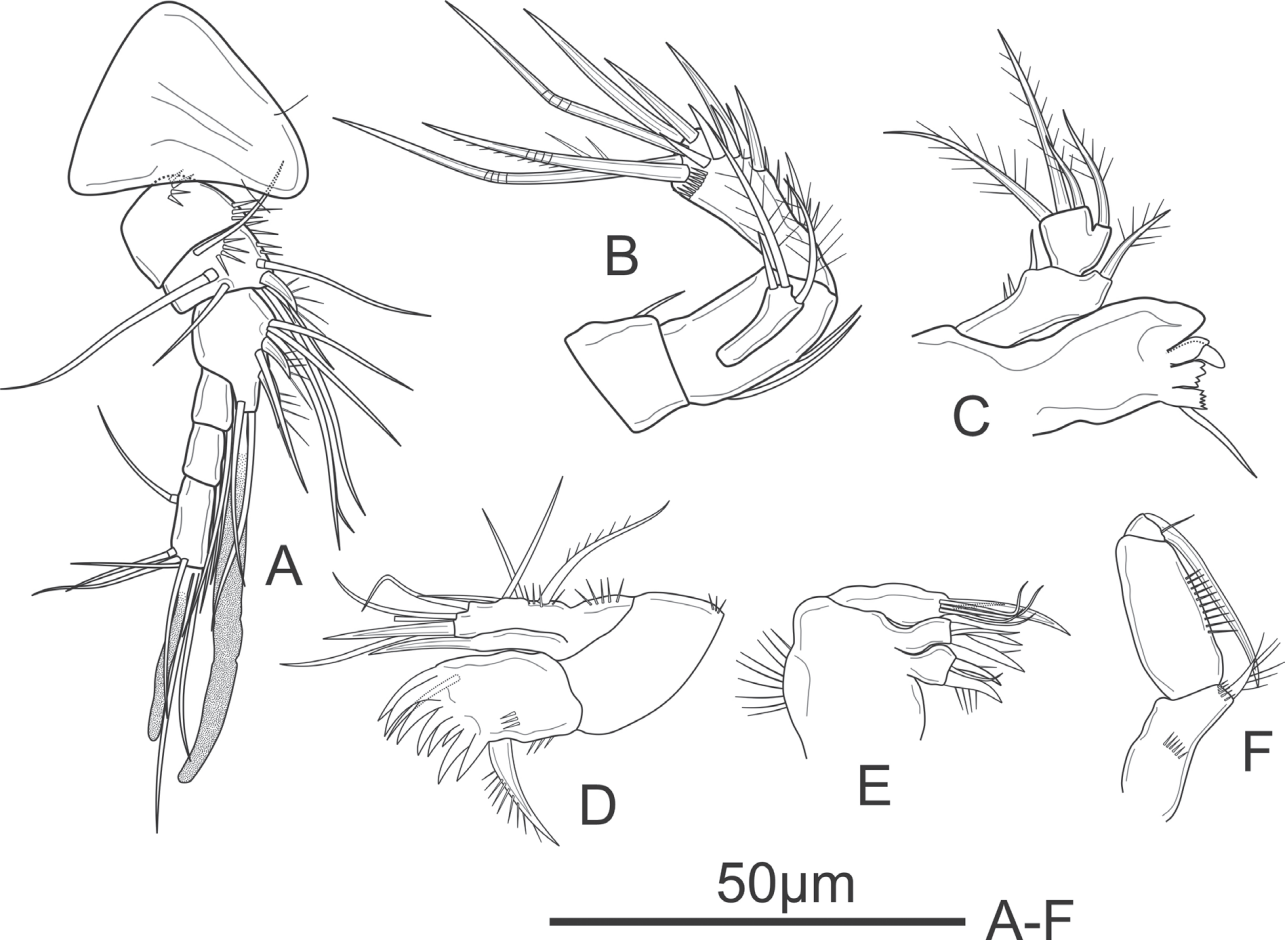
*Mesochra vietnamica* sp. nov. Female, holotype. A. Antennule and rostrum; B. Antenna; C. Mandible; D. Maxillule; E. Maxilla; F. Maxilliped.

**Figure 3.**
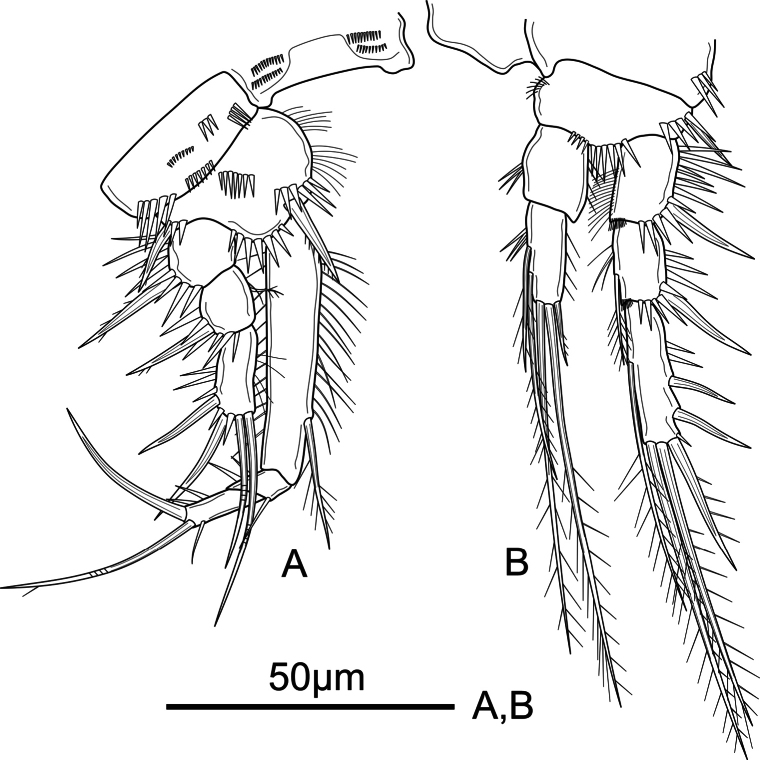
*Mesochra vietnamica* sp. nov. Female, holotype. A. P1; B. P2.

**Figure 4.**
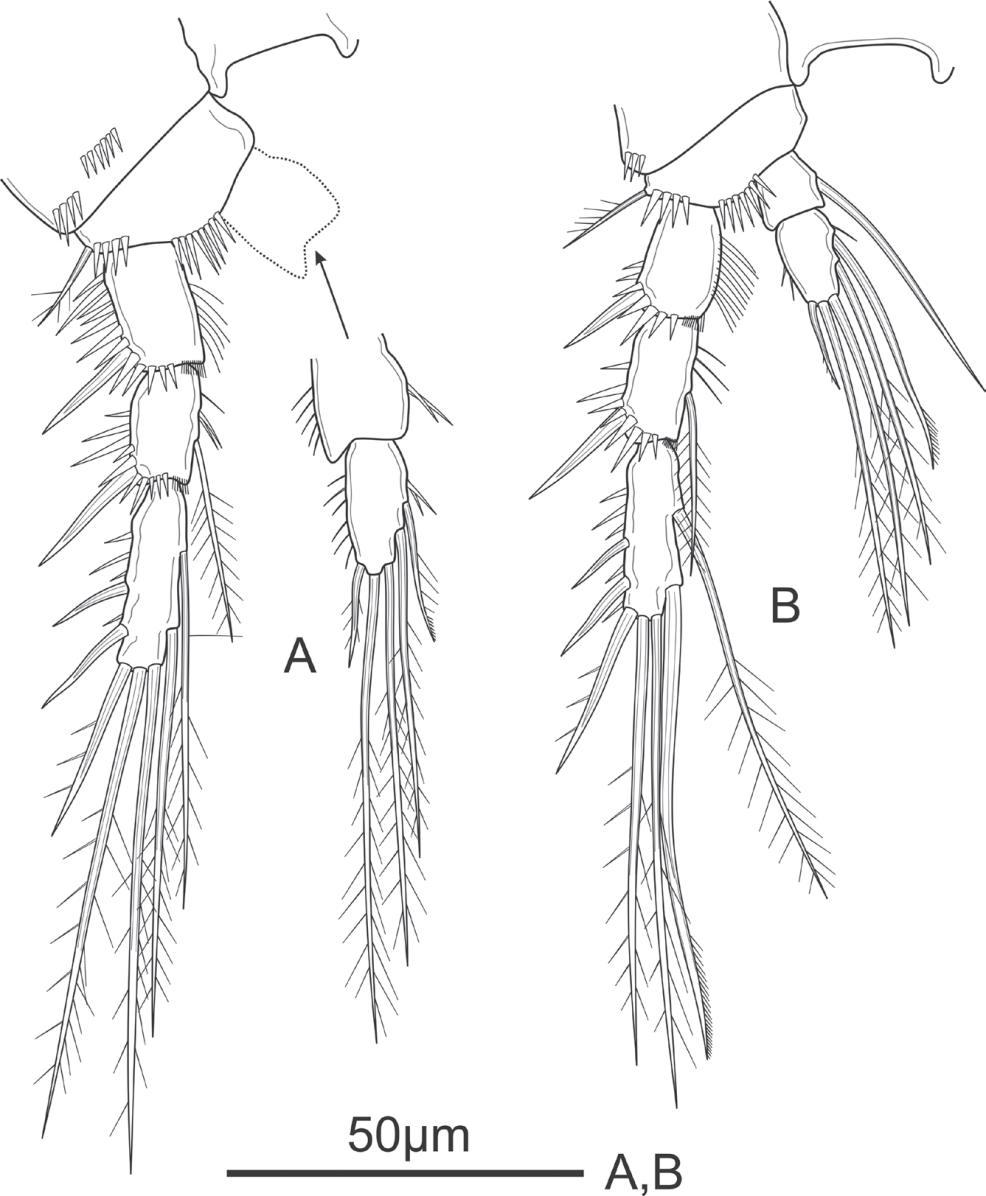
*Mesochra vietnamica* sp. nov. Female, holotype. A. P3; B. P4.

**Figure 5.**
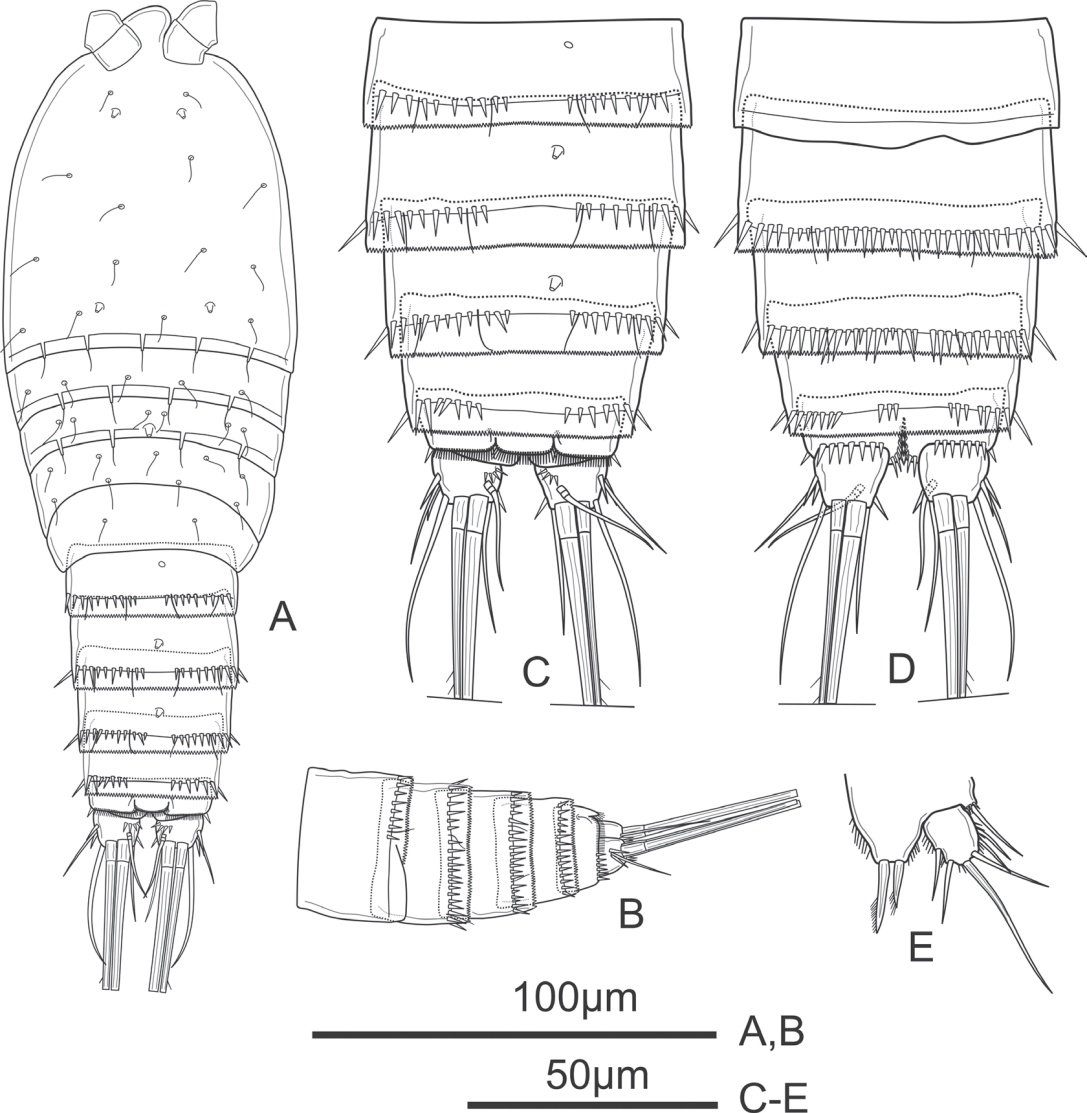
*Mesochra vietnamica* sp. nov. Male, allotype. A. Habitus, dorsal view; B. Urosome (P5-bearing somite omitted), lateral view; C. Urosome (P5-bearing somite omitted), dorsal view; D. Urosome (P5-bearing somite omitted), ventral view; E. P5.

**Figure 6.**
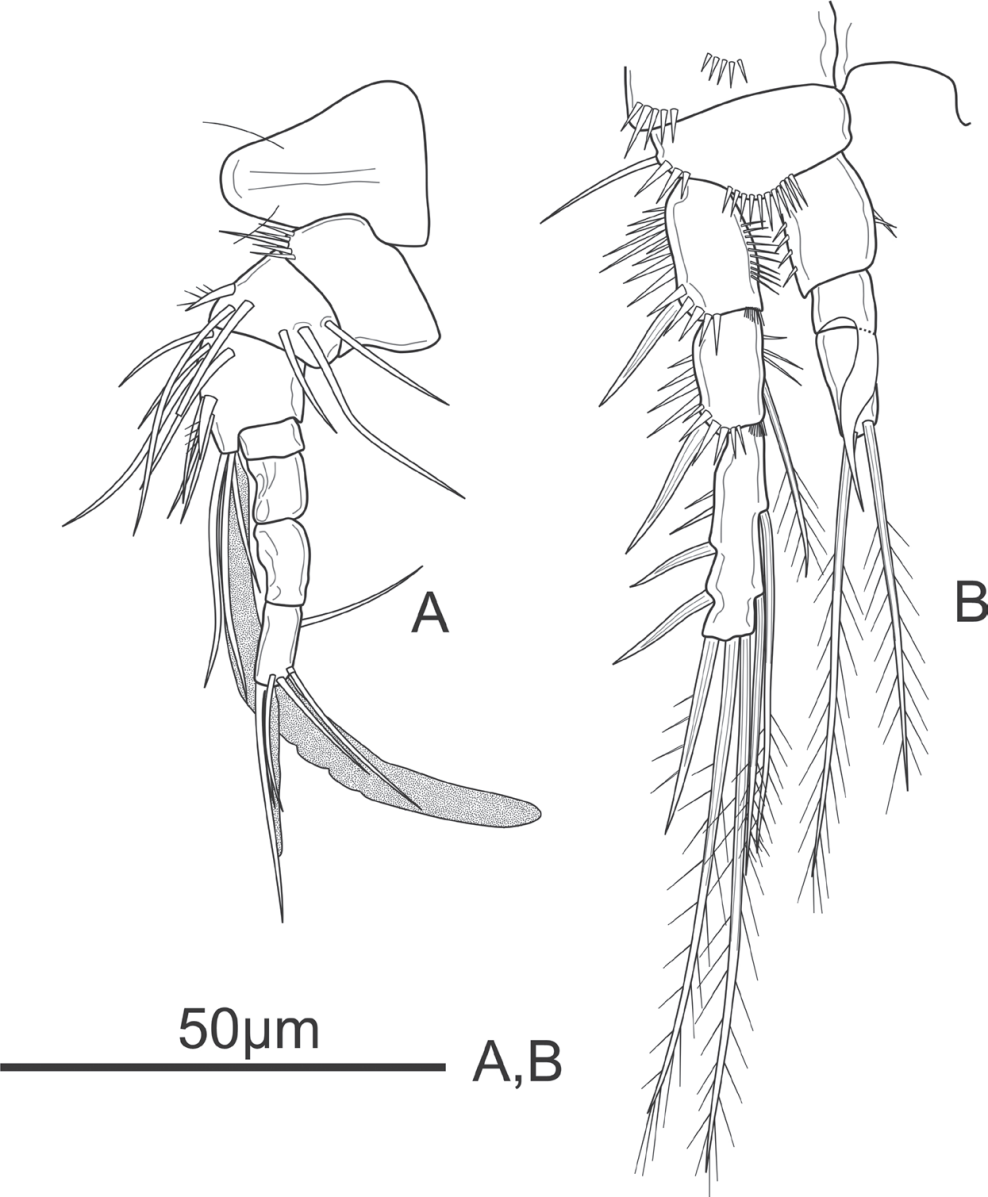
*Mesochra vietnamica* sp. nov. Male, allotype. A. Antennule and rostrum; B. P3.

Figs 1, 2, 3, 4, 5, 6

#### Material examined.

***Holotype***: adult female completely dissected and mounted on a slide (ZC-DTU-COPEPODA-0018). Allotype: adult male completely dissected and mounted on a slide (ZC-DTU-COPEPODA-0019). Paratypes: six females and three males stored in 70% alcohol (ZC-DTU-COPEPODA-0020). All material collected by N-S. Tran, Thi-Phuong Pham, Minh Anh Dam, Thi Hong Nguyen, Ngoc Dung Nguyen, and Van Da Phan on July 10, 2023.

#### Type locality.

Mangrove area with nipa palm (*Nypa fruticans*) along the Vu Gia–Thu Bon River (15°52'27"N, 108°22'36"E), Da Nang City, Central Vietnam; 0.5–2.0 m depth; muddy sand; salinity 23–27‰.

#### Etymology.

The specific epithet refers to the country where the new species was discovered. The name is an adjective in the nominative singular, gender feminine.

#### Differential diagnosis.

Female antennule 6-segmented, with principal aesthetasc on third segment. Antennary exopod with three setae. Maxillule with one strong seta on coxal endite and four lateral elements on coxobasis representing remnant of endopod and exopod; ventralmost unipinnate seta on praecoxal arthrite very strong and curved, being the largest element among those of the arthrite. P1 Enp 3-segmented; Enp-1 longer than exopod; Enp-2 with one inner seta. P2–P4 Exp-3 with three outer spines; P2–P4 Exp-3 with one inner seta. P2–P3 Enp-1 without inner seta. Female P2–P4 Enp-2 with five elements. Male P3 Enp 3-segmented. Female P5 Exp with five, male P5 Exp with six setae. Female and male P5 Exp and baseoendopod separated. Male P5 baseoendopods fused medially. Female P6 with two setae. Anal operculum fringed with small spinules.

#### Description.

**Female.** Habitus (Fig. 1A) fusiform, widest at the posterior part of the cephalothorax. Total body length measured from anterior margin of rostrum to posterior margin of caudal rami 238–288 µm (mean = 263 µm, *n* = 6; length of holotype = 261 µm). Rostrum (Figs 1A, 2A) large, broad, triangular, with rounded apex, with two subdistal lateral sensilla. Prosome gradually tapering from posterior part of cephalothorax to posterior part of P4-bearing somite; all prosomites with smooth posterior hyaline frill and with sensilla as shown. P5-bearing somite narrower than preceding somite, with smooth posterior hyaline frill and with fewer sensilla. Second (genital) and third urosomites fused dorsally forming genital double-somite (Fig. 1A, C, D), with ventrolateral vestige of division between somites (Fig. 1B, D); posterior half of genital double-somite with dorsal row of small spinules interrupted dorsally, with longer ornaments laterally, and with medial row of small spinules ventrally (Fig. 1B–D); anterior and posterior halves with dorsal pores as shown (Fig. 1C); genital complex (Fig. 1D) with narrow small copulatory pore medially, posterior to genital apertures. Fourth urosomite (Fig. 1B–D) resembles posterior half of genital double-somite. Fifth urosomite (Fig. 1B–D) without sensilla or spinules dorsally, with few long spinules laterally and two sets of three spinules ventrally. Anal somite (Fig. 1B–D) with row of small spinules dorsally and more robust ornamentation ventrally, and two spinules laterally; semicircular anal operculum with row of spinules along free margin and one sensilla on each side; ventrally cleft, with longitudinal spinules as shown. Caudal ramus (Fig. 1A–D) subquadrate, about as long as wide; with six elements as follows: anterolateral seta (I) absent, anterolateral seta (II) smooth, inserted at about ½ length of outer margin, flanked by two strong spinules; posterolateral seta (III) smooth, inserted in outer distal corner, about 2.6 times as long as caudal ramus; outer distal seta (IV) about 6.3 times as long as caudal ramus, unipinnate; inner terminal seta (V) strong, longest, pinnate; inner accessory seta (VI) thin and smooth, about 2.2 times as long as caudal ramus; dorsal seta (VII) about 1.5 times as long as caudal ramus, smooth, triarticulated, inserted at 2/3 of caudal ramus length, inserted close to inner margin.

Antennule (Fig. 2A) 6-segmented. First segment with row of spinules proximally and distally. Second segment with row of spinules dorsally and along inner margin. Second and third segments with one and two unipinnate spiniform setae, respectively. Aesthetasc on third segment reaching far beyond distal segment. Setal formula as follows: 0, 7, 9+ae, 1, 2, 5+acrothek.

Antenna (Fig. 2B) with coxa bearing one spinule. Allobasis 1.7 times as long as wide, with two abexopodal bare setae. Exopod 1-segmented, with three apical setae: one subdistal pinnate seta; apically with one pinnate and one bare seta different in length. Endopodal segment with spinules along inner margin proximally, two strong lateral spines and five elements apically: one geniculate pinnate and two smooth geniculate setae, and two smooth spines.

Mandible (Fig. 2C) with robust coxa bearing five strongly chitinized teeth and one seta. Mandibular palp 2-segmented; first basal segment with several spinules along outer margin, one pinnate seta on inner margin and one short spiniform exopodal seta; endopod with one pinnate seta on inner margin and apically with three pinnate setae unequal in length.

Maxillule (Fig. 2D) Praecoxal arthrite with one small surface seta, eight large robust spines apically, and one robust, unipinnate seta at ¾ of inner margin; with rows of spinules as shown. Coxa and basis fused; coxal endite with one spiniform seta and with outer row of spinules. Basis with proximal row of outer spinules, with two subdistal lateral setae, and two distal setae and one spiniform element apically; with four elements representing remnant of endopod and exopod.

Maxilla (Fig. 2E) composed of syncoxa and allobasis. Syncoxa with two endites; each endite with two spiniform and one smooth seta apically. Allobasis drawn out into bare, strong claw, with five smooth setae near its base (three of which probably endopodal).

Maxilliped (Fig. 2F) comprising syncoxa, basis, and Enp. Syncoxa with an anterior row of small spinules at ½ segment length, and row of small spinules at the base of spiniform unipinnate seta on inner margin apically. Basis about 2.2 times as long as wide, with longitudinal row of spinules along inner margin. Endopod with smooth, curved claw and a small accompanying seta.

P1–P4 (Figs 3A, B, 4A, B) with short unornamented intercoxal sclerites, except in P1 with two rows of small spinules on each side. The Exp/Enp length ratio for P1–P4 is 0.6, 1.7, 1.3, and 3.1, respectively.

P1 (Fig. 3A) coxa with four groups of small spinules unequal in size, and with spinules close to outer distal corner. Basis with anterior surface spinules, with stronger ornaments distally between rami, and at base of outer and inner spines, with setules along inner margin. Exopod 3-segmented; Exp-1 with one strong outer spine; Exp-2 with strong outer spine and slender pinnate inner seta; Exp-3 with two smooth outer spines and two geniculate distal setae. Endopod 3-segmented; Enp-1 reaching beyond Exp, with rows of spinules along inner and outer margin, with a pinnate inner seta inserted at distal third of segment; Enp-2 shortest, with one thin, smooth inner seta and two outer spinules; Enp-3 with three elements: one thin and smooth lateral inner seta, one geniculate inner distal seta, and one strong smooth outer distal spine.

P2 (Fig. 3B) coxa with few spinules close to outer distal corner. Basis with few strong spinules at the base of strong outer spiniform seta, and distally between rami, and with a transversal row of short, hair-like setules on inner margin. Exopod 3-segmented; segments with spinules on outer margin and setules along inner margin; Exp-1 with frill on distal inner corner and outer spine; Exp-2 with frill on distal inner corner, outer spine, and pinnate seta at 2/3 length of inner margin; Exp-3 with three outer spines of which two proximal smooth, distal one unipinnate, two pinnate seta apically and one at ½ length of inner margin. Endopod 2-segmented, reaching tip of Exp-2; segments with strong spinules along inner margin, and weak spinules along outer margin; Enp-1 unarmed; Enp-2 with five elements: two pinnate setae at ½ and ¾ length of inner margin, two long pinnate setae and one short spine apically.

P3 (Fig. 4A) coxa with one row of spinules anteriorly, and one on outer distal corner. Basis with spinules at the base of pinnate outer seta, and distally between rami. Exopod 3-segmented; Exp-1 and Exp-2 as in P2. Exp-3 as in P2 except for two pinnate inner setae. Endopod 2-segmented, almost reaching tip of Exp-2; Enp-1 as in P2; Enp-2 with five elements: one unipinnate and one pinnate inner seta, two pinnate setae apically, and one unipinnate outer spine.

P4 (Fig. 4B) coxa with spinules close to outer distal margin. Basis as in P3. Exopod 3-segmented; Exp-1 and Exp-2 as in P2 and P3; Exp-3 as in P3 except for subdistal inner seta visibly stronger and with distal fourth unipinnate. Endopod 2-segmented, reaching tip of Exp-1; Enp-1 unornamented, with one bare inner seta; Enp-2 with five elements: one stiff proximal and one subdistal seta on inner margin, two pinnate setae apically, and one unipinnate outer spine.

Armature formula of P1–P4 as in Table 2.

**Table 2.** Armature formula of swimming legs of *Mesochra vietnamica* sp. nov. (outer–inner seta/spine; outer–apical–inner seta/spine; Arabic numerals = number of setae, Roman numerals = number of spines).

Swimming leg	Basis	Exp			Enp		
		1	2	3	1	2	3
P1	I-I	I-0	I-1	II,2,0	0-1	0-1	0,I1,1
P2	I-0	I-0	I-1	III,2,1	0-0	I,2,2	
P3	1-0	I-0	I-1	III,2,2	0-0	I,2,2	
P4	1-0	I-0	I-1	III,2,2	0-1	I,2,2	

P5 (Fig. 1E) exopod and baseoendopod separated. Exopod with few spinules along inner and outer margin; with five elements: two short and smooth setae on outer margin, one smooth seta subapically, one long pinnate seta apically, one pinnate seta on inner margin; length ratio of the setae from inner to outer 1: 3.8: 1: 0.4: 0.6. Baseoendopod with few spinules on outer margin; with five unequal setae: two unipinnate proximal and one pinnate seta along inner margin subapically, and two pinnate setae apically.

P6 (Fig. 1D) represented by two setae of which inner smooth and outer pinnate.

**Мale.** General body shape (Fig. 5A) and ornamentation of urosomites (Fig. 5B–D) as in female. Total body length measured from anterior margin of rostrum to posterior margin of caudal rami 206–225 µm (mean = 215 µm, *n* = 3; length of allotype = 215 µm). Rostrum, anal operculum, and caudal rami as in female. Sexual dimorphism expressed in antennule, urosomal segmentation [second (genital) and third urosomites separated], P3 Enp, P5, and P6.

Antennule (Fig. 6A) 7-segmented. Surface of segments smooth except for a row of robust spinules on inner distal corner on first segment. All setae smooth, except for one spiniform seta on second and third segments. Aesthetasc on third segment long, reaching far beyond distal segment, and fused basally to slender seta. Setal formula as follows: 0, 6, 11+ae, 0, 0, 0, 4+acrothek.

P1, P2, P4 (not shown) as in female.

P3 (Fig. 6B) coxa, basis and exopod as in female Endopod 3-segmented; Enp-1 as in female. Enp-2 unornamented, with inner sinuous apophysis reaching beyond Enp-3, the latter with two pinnate setae apically of which outer slightly longer.

P5 (Fig. 5E) exopod and baseoendopod separated; baseoendopod of both legs fused medially. Exopod with spinules along inner margin; with six smooth unequal setae; length ratio of setae from inner to outer 1: 1.2: 3: 1.6: 0.9: 0.9. Baseoendopod with spinules along inner and outer margin; with two spiniform unipinnate setae apically, of which outer shorter.

P6 (Fig. 5D) represented by smooth simple plate; fused to somite.

## ﻿Discussion

The material presented herein was attributed to the genus *Mesochra* based on the combination of: i) the semicircular anal operculum with small spinules along free margin, ii) the short caudal rami, iii) the 6-segmented female antennule, iv) the well-developed, distinct rostrum, v) the 1-segmented antennary exopod with three setae, vi) the 3-segmented P1 Exp and Enp; vii) the P1 Enp-1 longer than exopod; viii) the presence of four setae/spines on P1 EXP-3; ix) the 3-segmented female and male P2–P4 Exp; x) the 2-segmented female and male P2 and P4 Enp; xi) the 2-segmented female P3 Enp, sexually dimorphic (3-segmented with an apophysis on Enp-2) in the male; xii) the P5 Exp and baseoendopod separated in the female and male; xiii) the P5 baseoendopods separated in the female, but fused medially in the male.

The new species out keys as a member of *Mesochra* in the keys by Lang (1948), Hamond (1971), Fiers and Rutledge (1990), and Wells (2007). Six additional species were described after the publication of Wells’ (2007) monograph. These are *M. oligochaeta* Kornev & Chertoprud, 2008, *M. ingolfsoni* Gómez & Steinarsdóttir, 2007, *M. snoppa* Gómez & Steinarsdóttir, 2007, *M. freyri* Gómez & Steinarsdóttir, 2007. *M. bisetosa* Lee & Chang, 2008, and *M. huysi* Suárez-Morales & Fuentes-Reinés, 2015. The new species seems to be morphologically similar to *M. stellfeldi* Jakobi, 1954, *M. nana* Brady, 1910, *M. pygmaea*, *M. bodini* Kunz, 1975, *M. oligochaeta*, *M. ingolfsoni*, *M. snoppa*, and *M. freyri* in the combination of i) the ornamentation of the anal operculum (with small spinules along free margin); ii) the well-developed rostrum; iii) the 6-segmented female antennule; iv) the presence of an inner seta on P1 Enp-2; v) the 3-segmented P1 Enp, with Enp-1 longer than the entire Exp; vi) the presence of three outer spines on P2–P4 Exp-3; vii) the presence of one inner seta on P2 Exp-3; viii) the presence of five setae/spines on P2–P4 Enp-2; ix) the presence of five elements on the female P5 baseoendopod; and x) the separated P5 Exp and baseoendopod in both sexes. However, the new species is different from all these species in the relative situation of the inner seta on P1 Enp-1. This seta is situated in the distal third in the new species, but midway the inner margin in *M. stellfeldi*, *M. nana*, *M. pygmaea*, *M. oligochaeta*, and *M. ingolfsoni*, slightly below the middle of the segment in *M. bodini* and *M. freyri*, or in the proximal third in *M. snoppa* (Table 3). Furthermore, the new species is different from *M. stellfeldi*, *M. nana*, *M. pygmaea*, *M. bodini*, *M. ingolfsoni*, *M. snoppa*, and *M. freyri* in the armature of P2–P3 Enp-1 (unarmed in the new species, but with one seta in the other seven species).

**Table 3.** Comparison of characters among eight close-related species of the genus *Mesochra* (the superscripts denote the corresponding reference).

Characters	*M. vietnamica* sp. nov.	*M. stellfeldi* Jakobi, 1954	*M. pygmaea* (Claus, 1863)	*M. nana* Brady, 1910	*M. bodini* Kunz, 1975	*M. ingolfsoni* Gómez & Steinarsdóttir, 2007	*M. snoppa* Gómez & Steinarsdóttir, 2007	*M. freyri* Gómez & Steinarsdóttir, 2007	*M. oligochaeta* Kornev & Chertoprud, 2008
**Female**									
Shape of rostrum	Triangular	NA	NA	NA	Broad, bent downwards	Bell-shaped	Bell-shaped	Rectan-gular	Triangular
Ornamentation of free margin of anal operculum	Fine spinules (25–27)	NA	Fine spinules^*^ (?)	Naked^*^	Fine spinules(~20)	Fine spinules(27^†^)	Fine spinules(25^†^)	Fine spinules(17^†^)	NA
Number of segments of antennule	6	6-7	6	7^**^	6	6	6	6	6
Number of pinnate elements on segment II of the antennule	1	NA	NA	NA	NA	2	2	5	NA
Number of elements on the exopod of the antenna	3	4	3	3^*^	3	3	3	3	2
Characteristic of ventralmost element on praecoxal arthrite of maxillule	Strong and curved pinnate element	NA	NA	NA	NA	Short and straight pinnate element	Short and slightly curved smooth element	Short and slightly curved smooth element	Short and slightly curved element
Length/width ratio of P1 Enp-1	4.7	6.6^†^	4.4^†***^ or 5.1^†****^	7.3^†*^ or 4.7^†**^	5.5	5.3	5.3	6.6	>8.5
Length ratio of P1 Enp-1 to Exp^†^	1.3	1.3	1.3^***^	1.3^*^ or 1.5^**^	1.3	1.3	1.6	1.4	1.3
Length/width ratio of P1 Enp-2^†^	1.7	1	1.5^***^	1.5^*^ or 1^**^	1.5	1	1.5	2	1.5
Length ratio of P1 Enp-3 to Enp-2^†^	1.4	2	2^***^	1.5^*^ or 1^**^	2.5	1.5	1.5	2	1.5
Number of inner setae on P1 Enp-1 and Enp-2	1/1	1/1	1/1	0/0^**^ or 1/1^*^	1/1	1/1	1/1	1/1	1/1
Insertion point of inner seta of P1 Enp-1	Distal third	Middle	Middle	Middle	Proximal third	Middle	Proximal third	Middle	Middle
Number of setae/spines on P2–P4 Exp-3	6.7.7	6.7.7	6.7.7^***^	6.7.7*	5-6.7.7	6.7.7	6.7.7	6.7.7	6.7.7
Number of setae/spines on P2–P4 Enp-1	0.0.1	?.1.?	1.1.1^***^	1.1.1^*^	1.1.1	1.1.1	1.1.1	1.1.1	0.0.1
Number of setae/spines on P2–P4 Enp-2	5.5.5	5.5.5	5.5.5	5.5.5^*^	5.5.5	5.5.5	5.5.5	5.5.5	4.5.5
Number of elements on P5 Exp and baseoendopod	5/5	5/5	5/5	5/5	5/5	5/5	5/5	5/5	5/5
Number of elements on the P6	2	NA	NA	NA	NA	2	3	1	1^†^
**Male**									
Number of elements on the P5 baseoendopod and Exp	2/6	2/5	2/5	NA	2/6	2/6	2/6	2/6	2/6
Number of segments of P3 Enp	3	2	3	NA	2	3	3	3	3
Length of the apophysis relative to Enp-3	Longer	Shorter	Longer	NA	NA^‡^	Longer	Longer	Longer	Equal

*According to Lang (1948); **According to Brady (1910); ***According to Sars (1905); ****According to Marinov and Apostolov (1985); NA^‡^Kunz (1975) described the male third swimming leg has only two segments and lacks apophysis. Wells (2007) argued that it is likely incorrect. ^†^Estimated based on the length measured or the number counted from the illustration presented in the contribution.

*Mesochra vietnamica* sp. nov. is morphologically close to *M. oligochaeta* described from White Sea by the absence of an inner seta on P2–P3 Enp-1, the armature complement of P1–P4 Exp and the number of elements on the endopodal lobe and Exp of P5 in both sexes, but they differ in the length/width ratio of P1 Enp-1 (about 10 times as long as wide in *M. oligochaeta*, but 4.6 times as long as wide in the new species) and, as noted above, in the position at which the inner seta on P1 Enp-1 is inserted (at about mid-segment in *M. oligochaeta*, but in the distal third in *M. vietnamica* sp. nov.), number of elements on the antennary exopod (two in *M. oligochaeta*, but three in the new species), and number of elements on P2 Enp-2 (four in *M. oligochaeta*, but five in the new species).

Regarding the relative length of P1 Enp-1, members of the genus *Mesochra* can be categorized into three distinct groups of species based on the length of the segment relative to the length of the entire Exp. The first group, including the new species, is characterized by P1 Enp-1 being longer than the entire Exp. This is the largest group within the genus, comprising species such as *M. nana*, *M. stellfeldi*, *M. bodini*, and several other related congeners. The second group consists of species in which P1 Enp-1 is slightly longer than wide and reaches the tip or slightly beyond the tip of the Exp-1. This group includes *M. bisetosa*, *M. hinumaensis* Kikuchi, 1972, and *M. quadrispinosa* Shen & Tai, 1965. The last group comprises species in which P1 Enp-1 is as long as, or longer than Exp-1 and Exp-2 combined but extending only up to the tip of Exp-3. Representatives of this group include *M. aestuarii* Gurney, 1921, *M. baylyi* Hamond, 1971, *M. inermis* (Brady, 1902), *M. lilljeborgii* Boeck, 1865, *M. lybica* Blanchard & Richard, 1891, *M. robertsoni* Brady, 1880, *M. xenopoda* Monard, 1930, *M. rapiens* (Schmeil, 1894), *M. inconspicua* (Scott, 1895), *M. heldti* Monard, 1935, *M. flava* Lang, 1933, *M. arenicola* Nicholls, 1939, *M. pestai* Lang, 1948, *M. mexicana* Wilson, 1971, and *M. pallaresae* Soyer, 1977.

Among the differential diagnostic characteristics of the new species and *M. oligochaeta*, three are present in only a limited number of accepted taxa of the genus. These include: i) the inner seta positioned in the distal third of P1 Enp-1, rather than on proximal half, in the middle, or slightly below the middle of the segment; ii) the presence of two elements on the antennary exopod, in contrast to the typical trisetose condition, and iii) the presence of four elements on P2 Enp-2, instead of the usual presence of five elements. The following species exemplify the occurrence of the aforementioned characteristics:

- Inner seta of P1 Enp-1 situated at the distal third: *M. aestuarii*, *M. baylyi*, *M. inermis*, *M. lilljeborgii*, *M. lybica*, *M. pacifica*, *M. pestai*, *M. robertsoni*, *M. xenopoda*, *M. alaskana* Wilson, 1958, and *M. pontica* Marcus, 1965;
- Antennary exopod bearing only two elements: *M. schmidti* Mielke, 1974;
- P2 Enp-2 bearing four elements: *M. hinumaensis*, and *M. xenopoda*.

Notably, *Nannomesochra arupinensis* (Brian, 1925)—originally known as member of *Mesochra*—along with the other species within *Nannomesochra*, also exhibit the reduced number of elements (two) on the antennary exopod. However, representatives of the genus *Mesochra* can be differentiated from those of *Nannomesochra* by the 2-segmented mandibular palp.

### ﻿Key to species of *Mesochra* recorded in Asia

A key to the species of *Mesochra* known from Asia is herein presented to facilitate future identifications. *Mesochra prowazeki* was included based on the description by Shen et al. (1979).

#### ﻿Key to the females of the species of Mesochra recorded in Asia

**Table:** 

1	P1 Enp 3-segmented	**2**
–	P1 Enp 2-segmented	**10**
2	P2–P4 Exp-3 with two outer spines; P1 Enp-2 shorter than wide	***M. prowazeki* Douwe, 1907**
–	P2–P4 Exp-3 with three outer spines; P1 Enp-2 as long as wide or longer than wide	**3**
3	P1 Enp-1 short, reaching slightly beyond Exp-1 or reaching the apical margin of Exp-3 at the most	**4**
–	P1 Enp-1 longer than Exp	**7**
4	P1 Enp-1 reaching slightly beyond Exp-1; female P2–P4 Enp with three or four elements on Enp-2; female P5 baseoendopod with four setae	**5**
–	P1 Enp-1 reaching almost to the apical margin of Exp-3; female P2–P4 Enp with five elements on Enp-2; female P5 baseoendopod with five or six elements	***M. rapiens* (Schmeil, 1894)**
5	P5 Exp fused to baseoendopod, with five elements	**6**
–	P5 Exp not fused to baseoendopod, with four elements	***M. quadrispinosa* Shen & Tai, 1965**
6	P2–P4 Enp-2 with 4.4.4 setae	***M. hinumaensis* Kikuchi, 1972**
–	P2–P4 Enp-2 with 3.3.4 setae	***M. bisetosa* Lee & Chang, 2008**
7	P1 Exp-2 without inner seta	**8**
–	P1 Exp-2 with inner seta	**9**
8	Anal operculum with fine spinules	***M. pygmaea* (Claus, 1863)**
–	Anal operculum unornamented	***M. nana* Brady, 1910**
9	Inner seta on P1 Exp-2 short, reaching almost to the apical margin of Exp-2; P2 and P3 Enp-1 without and P4 Enp-1 with inner seta; P5 Exp and endopodal lobe with five setae	***M. vietnamica* sp. nov.**
–	Inner seta on P1 Exp-2 reaching beyond to the apical margin of Exp-3; P2–P4 Enp-1 with one inner seta; P5 Exp with five, endopodal lobe with six setae	***M. alaskana* Wilson, 1958**
10	P5 Exp fused to baseoendopod; P5 exopodal lobe with four, endopodal lobe with five setae	***M. wolskii* Jakubisiak, 1933**
–	P5 Exp not fused to baseoendopod	**11**
11	P1 Enp-2 with two elements (claw-like spine, and geniculate seta), without inner seta	**12**
–	P1 Enp-2 with three elements (claw-like spine, geniculate seta, and short slender seta)	**13**
12	P2–P3 Enp-1 without inner seta	***M. suifunensis* Borutzky, 1952 sensu Borutzky**
–	P2–P3 Enp-1 with inner seta	***M. sewelli* Lang, 1948**
13	P5 Exp with four or five elements of which innermost seta longest	14
–	P5 Exp with five elements of which innermost seta shorter than the next element nearby	**15**
14	Inner seta on P1 Enp-1 issuing at the distal half of segment; anal operculum with marginal small spinules	***M. suifunensis* Borutzky, 1952 sensu Shen et al.**
–	Inner seta on P1Enp-1 issuing at the middle of segment; anal operculum smooth	***M. meridionalis* Sars, 1905**
15	P2–P4 Exp-3 with 4.6.6 setae; exopod of mandible with three setae	***M. aestuarii* Gurney, 1921**
–	P2–P4 Exp-3 with 5.6.6 setae; exopod of mandible with four setae	***M. aralensis* Borutzky, 1927**

#### ﻿Key to the males of the species of *Mesochra* recorded in Asia

**Table:** 

1	P1 Enp 2-segmented; P2–P4 Exp-3 with two outer spines	**2**
–	P1 Enp 3-segmented; P2-P4 Exp-3 with three outer spines	**7**
2	P5 Exp fused to baseoendopod; P5 exopodal lobe with five or six, endopodal lobe with two setae; apophysis of P3 Enp-2 reaching tip of Enp-3 at the most	***M. wolskii* Jakubisiak, 1933**
–	P5 Exp not fused to baseoendopod; P3 Enp-2 with or without apophysis	**3**
3	P3 Enp-2 without apophysis	**4**
–	P3 Enp-2 with apophysis	**5**
4	P5 Exp fused to baseoendopod, with six elements	***M. meridionalis* Sars, 1905**
–	P5 Exp not fused to baseoendopod, with five elements	***M. suifunensis* Borutzky, 1952 sensu Borutzky**
5	P5 Exp with six, baseoendopod with three setae; P3 Enp-1 with inner seta; apophysis of P3 Enp-2 reaching beyond Enp-3	**6**
–	P5 Exp with six, baseoendopod with two setae; P3 Enp-1 without inner seta; apophysis reaching almost to the apical margin of Enp-3	***M. suifunensis* Borutzky, 1952 sensu Shen et al.**
6	P2–P4 Exp-3 with 4.6.6 setae; exopod of mandible with three setae	***M. aestuarii* Gurney, 1921**
–	P2–P4 Exp-3 with 5.6.6 setae; exopod of mandible with four setae	***M. aralensis* Borutzky, 1927**
7	P1 Enp-1 short, reaching slightly beyond Exp-1 or reaching the apical margin of Exp-3	**8**
–	P1Enp-1 longer than Exp	**11**
8	P1 Enp-1 reaching slightly beyond Exp-1; P2 and P4 Enp-2 with three or four setae; P3 Exp normally developed (unmodified)	**9**
–	P1 Enp-1 reaching almost to the apical margin of Exp-3; P2 and P4 Enp-2 with five setae; P3 Exp modified into robust and prehensile appendage	***M. rapiens* (Schmeil, 1894)**
9	P2 Enp-2 with three, P4 Enp-2 with four setae	***M. bisetosa* Lee & Chang, 2008**
–	P2 and P4 Enp-2 with four setae	**10**
10	Innermost element of P5 Exp relatively long, reaching beyond the tip of the next outer element	***M. hinumaensis* Kikuchi, 1972**
–	Innermost element of P5 Exp relatively short, reaching just about middle of the next outer element	***M. quadrispinosa* Shen & Tai, 1965**
11	P5 Exp with four, endopodal lobe with three setae	***M. alaskana* Wilson, 1958**
–	P5 Exp with five, endopodal lobe with two setae	**12**
12	P1 Enp-1 with inner seta on distal third; P2 Enp-1 without inner seta; P5 Exp with six setae	***M. vietnamica* sp. nov.**
–	P1 Enp-1 with inner seta at the middle P2 Enp-1 with inner seta; P5 Exp with five setae	***M. pygmaea* (Claus, 1863)**

## Supplementary Material

XML Treatment for Mesochra vietnamica

## ﻿Additional information

### Use of AI

No use of AI was reported.

### Author ORCIDs

Chaichat Boonyanusith https://orcid.org/0000-0003-1487-2160

Thi-Phuong Pham https://orcid.org/0009-0003-4203-6183

Minh Anh Dam https://orcid.org/0009-0000-3123-3884

Ngoc-Son Tran https://orcid.org/0000-0003-2387-5822

Samuel Gómez https://orcid.org/0000-0002-8597-8846

### Data availability

All of the data that support the findings of this study are available in the main text.
